# Anchoring Fe_3_O_4_ nanoparticles in a reduced graphene oxide aerogel matrix via polydopamine coating

**DOI:** 10.3762/bjnano.9.55

**Published:** 2018-02-15

**Authors:** Błażej Scheibe, Radosław Mrówczyński, Natalia Michalak, Karol Załęski, Michał Matczak, Mateusz Kempiński, Zuzanna Pietralik, Mikołaj Lewandowski, Stefan Jurga, Feliks Stobiecki

**Affiliations:** 1 NanoBioMedical Centre, Adam Mickiewicz University, Umultowska 85, 61-614 Poznań, Poland; 2 Institute of Molecular Physics, Polish Academy of Sciences, M. Smoluchowskiego 17, 60-179 Poznań, Poland; 3 Faculty of Physics, Adam Mickiewicz University, Umultowska 85, 61-614 Poznań, Poland

**Keywords:** aerogel, composite, Fe_3_O_4_ nanoparticles, polydopamine, reduced graphene oxide

## Abstract

Reduced graphene oxide–magnetite hybrid aerogels attract great interest thanks to their potential applications, e.g., as magnetic actuators. However, the tendency of magnetite particles to migrate within the matrix and, ultimately, escape from the aerogel structure, remains a technological challenge. In this article we show that coating magnetite particles with polydopamine anchors them on graphene oxide defects, immobilizing the particles in the matrix and, at the same time, improving the aerogel structure. Polydopamine coating does not affect the magnetic properties of magnetite particles, making the fabricated materials promising for industrial applications.

## Introduction

Preparation of hybrid aerogels based on two-dimensional carbon nanomaterials with unique physicochemical properties is among the most popular recent nanotechnological trends [1]. With this respect, graphene oxide (GO) is one of the most exploited aerogel-forming nanomaterials, as it allows obtaining three-dimensional highly porous structures, characterized by low density and high specific surface area [2–5]. Typically, the fabrication of aerogel is a multistep process, involving hydrogel formation via sol–gel technique followed by freeze drying or critical point drying [6]. During hydrogel formation, GO undergoes reduction. Therefore, after solvent removal, it forms a reduced graphene oxide (rGO) porous structure [7]. Currently, lots of research has been focused on the potential applications of rGO-based aerogels in energy storage systems (i.e., Li batteries [8–11], supercapacitors [12–16]), sensors (gas sensors [17–19], biosensors [20–21]) and adsorbers (oil pollution [22–23], organic contaminants [24–25]). Moreover, the properties of GO-based aerogels can be modified by addition of various functional additives, e.g., nanoparticles or polymers [26–29]. As produced hybrid aerogels are featured by unique properties with wide area of new and interesting potential applications [30–32]. One of the most interesting hybrids are those with magnetic properties, as these can be used as magneto-responsive adsorbers [33–34], gas sensors [18] or even nanoswitches/actuators [22]. The magnetic properties of hybrid aerogels are related to the presence of magnetic nanoparticles (MNPs) which can be in ferromagnetic or superparamagnetic state and are embedded in aerogel matrix. Iron oxide nanoparticles, such as magnetite (Fe_3_O_4_) or maghemite (γ-Fe_2_O_3_), are common functional additives widely applied in many different branches of science [35–36]. This is mainly thanks to their low price, simplicity of production, biocompatibility and environmental friendliness. There are two commonly used methods of iron oxide MNPs introduction into aerogel. The first method is based on the addition of iron precursors to GO water dispersion and “in situ” synthesis of iron oxide MNPs during hydrothermal hydrogel formation in autoclave. The precipitated nanoparticles are anchored to GO structure via Fe–C–O bonds or confined between GO sheets [11,16,18,33–34]. This method is based on a simple single-step process; however, its main drawback is the lack of the control over nanoparticle synthesis. Therefore, as prepared iron oxide MNPs are often agglomerated (form ferromagnetic agglomerates) and heterogeneous in size and shape. The second method involves the addition of already prepared nanoparticles to GO water dispersion before hydrogel formation [22]. As introduced MNPs physically adsorb at GO defects or unsaturated edges and become trapped in between GO nanosheets. The main advantage of this approach is the homogeneity in size, shape and oxidation state of MNPs, all of which influence the magnetic properties. The preparation of homogeneous MNPs often requires the use of hydrophobic stabilizers, such as oleic acid, which protect the material from environmental oxidation [37]. Interestingly, in several published articles iron oxide MNPs were stabilized via organic polymers, such as polydopamine (PDA) [38–39], which introduce additional functional groups onto the nanoparticles surface. Polydopamine is a synthetic analogue of melanine which is composed of dihydroxyindole, indoledione and dopamine units that are believed to be linked covalently [40]. Recently, scientific attention was turned to PDA nanocomposites, including those with GO and magnetite [41]. However, there are no reports related to the formation of hybrid aerogels based on the aforementioned compounds. In this work, GO-based aerogels with embedded PDA-coated magnetite nanoparticles were prepared and the physicochemical properties of this hybrid system were studied. The investigations were focused on the influence of the involved constituents and their cross-interactions on the properties of the composite. It was found that coating the magnetite particles with polydopamine (PDA) anchors the particles on graphene oxide defects, immobilizes them in the porous matrix, improves the aerogel structure and, what is important, do not affect the magnetic properties of magnetite.

## Results and Discussion

**Figure 1:**
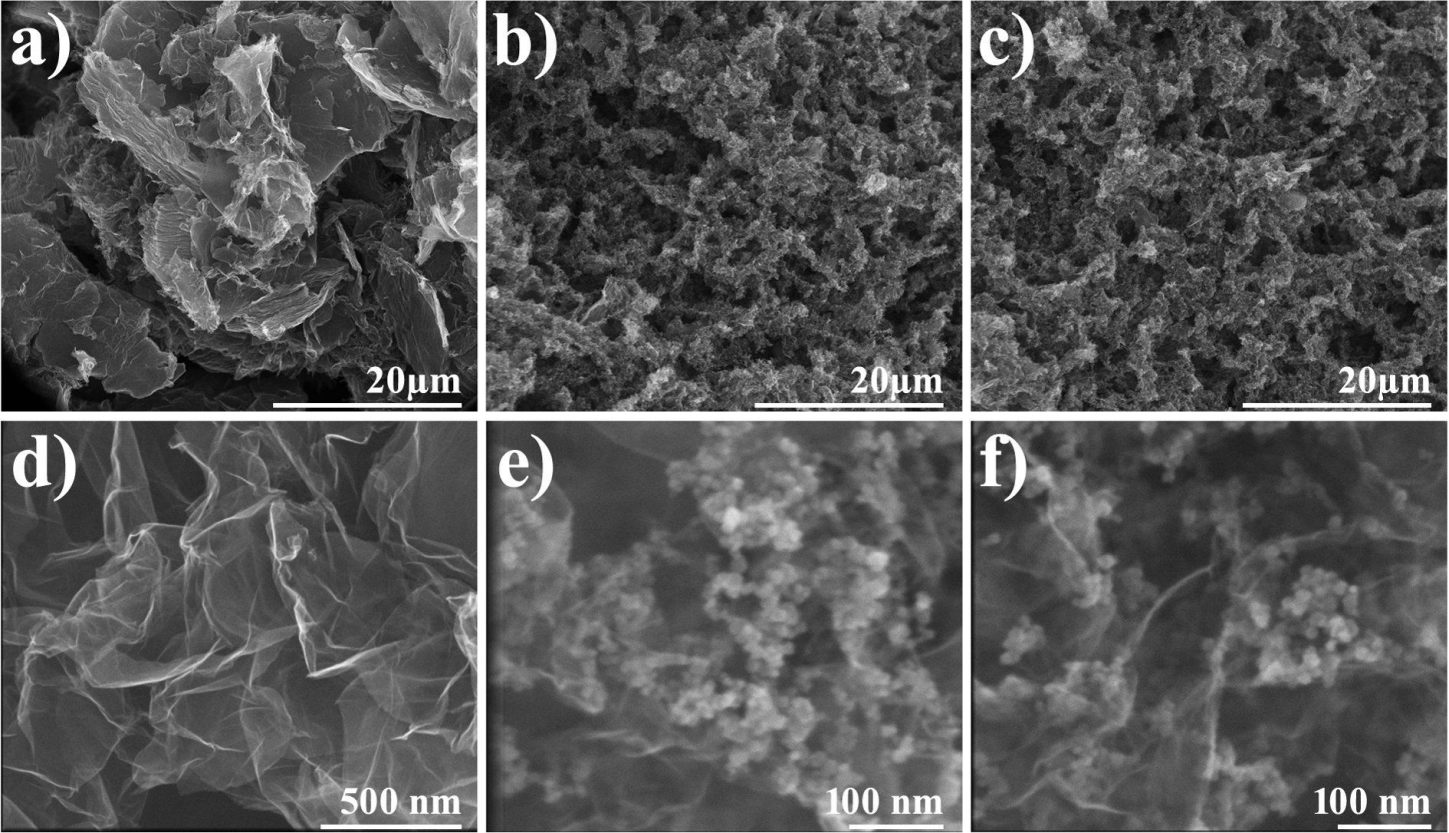
SEM micrographs of rGO (a,d), rGO-Fe_3_O_4_ (b,e) and rGO-PDA@Fe_3_O_4_ (c,f) aerogel structures.

**Figure 2:**
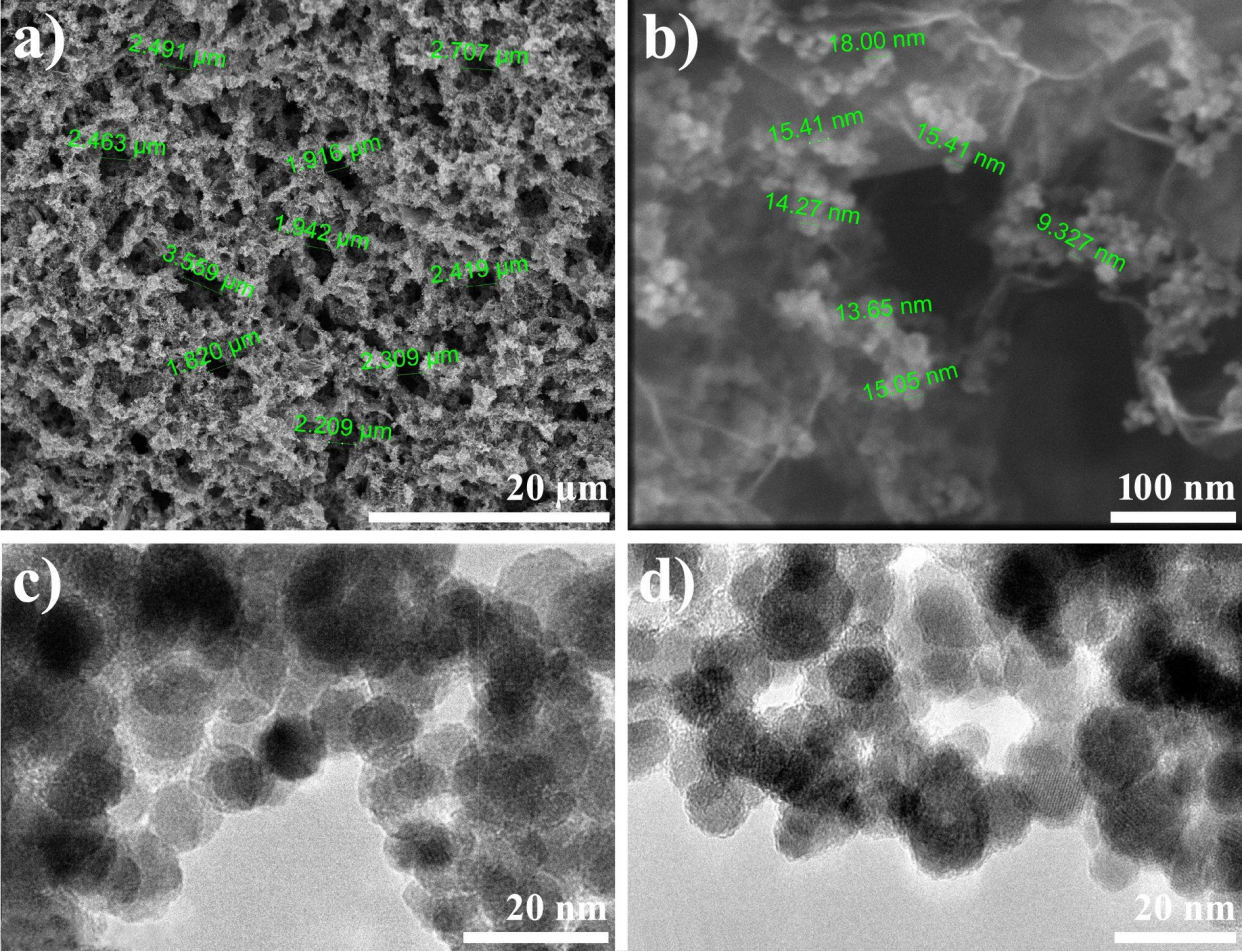
SEM micrographs of rGO-PDA@Fe_3_O_4_ aerogel (a,b) and TEM micrographs of Fe_3_O_4_ (c) and PDA@Fe_3_O_4_ nanoparticles (d).

The rGO, rGO-Fe_3_O_4_ and rGO-PDA@Fe_3_O_4_ aerogels were synthesized as described in the Experimental section. The crystallographic structures of pure constituents, i.e., GO and Fe_3_O_4_, were confirmed by XRD and is presented in Figure S1 of Supporting Information File 1 [42]. The morphology of the prepared aerogel samples was studied using SEM and TEM. Figure 1 presents SEM micrographs of rGO, rGO-Fe_3_O_4_ and rGO-PDA@Fe_3_O_4_ aerogels. From the low magnification images one can determine the pore size distribution and observe interconnected 3D network of aerogel-forming rGO structures (Figure 1a–c). At higher magnification, the agglomerates of MNPs are clearly visible (Figure 1e,f). From this figure it can be deduced that the distribution of agglomerated MNPs is homogeneous on both sides of the rGO sheets. Figure 2 presents selected SEM micrographs with marked pore size distribution (Figure 2a) and diameter of MNPs (Figure 2b) in rGO-PDA@Fe_3_O_4_ aerogel, as well as TEM micrographs of MNPs in rGO-Fe_3_O_4_ and rGO-PDA@Fe_3_O_4_ samples (Figure 2c,d). The analyzed pore sizes are in the range of 1.5 μm to 3.5 μm. The diameter distribution of magnetite nanoparticles was homogenous in all samples (9 nm to 18 nm). From Figure 2d one can observe that regardless of PDA coating, the Fe_3_O_4_ nanoparticles are present in agglomerated form.

**Figure 3:**
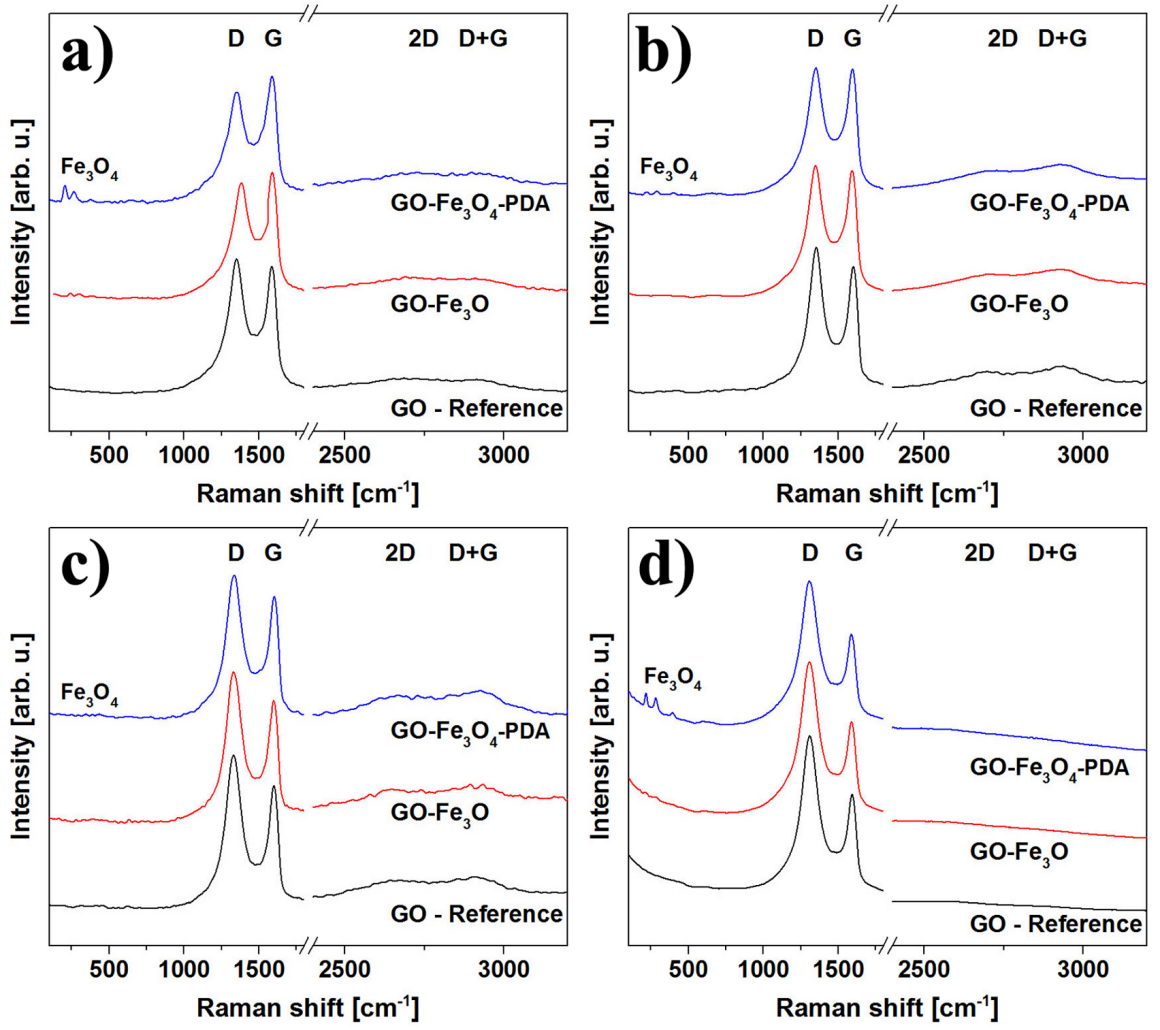
Raman spectra of rGO, rGO-Fe_3_O_4_ and rGO-PDA@Fe_3_O_4_ aerogel samples: λ = 488 nm (a), 514 nm (b), 633 nm (c) and 785 nm (d).

**Figure 4:**
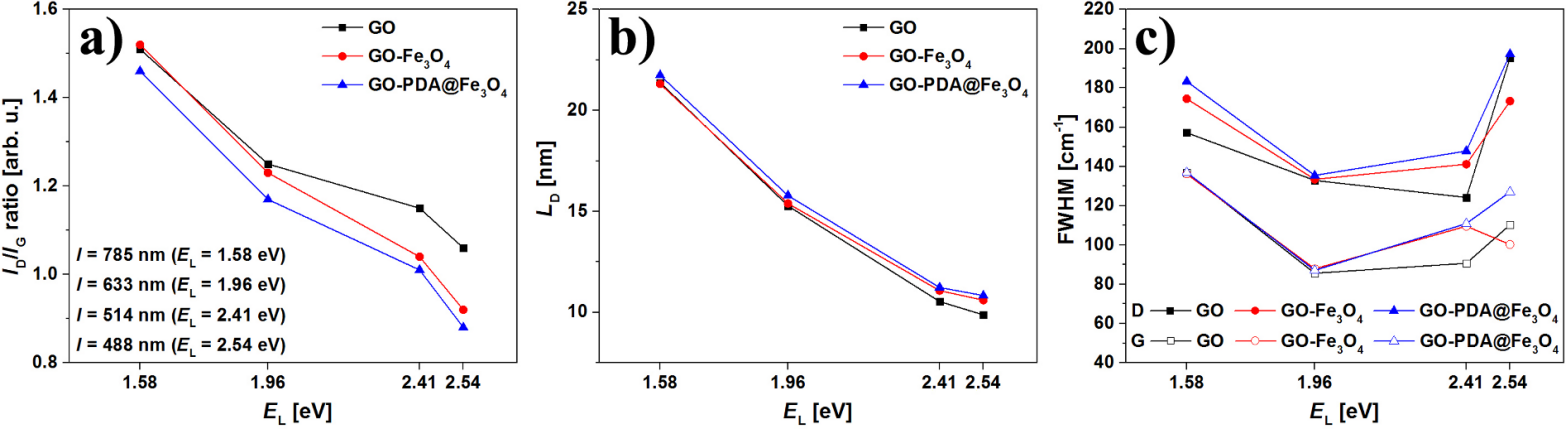
*I*_D_/*I*_G_ ratios (a), mean defect distance (*L*_D_) (b) and FWHM (c) calculated from Raman spectra of rGO, rGO-Fe_3_O_4_ and rGO-PDA@Fe_3_O_4_ aerogel samples.

The vibrational properties of the prepared aerogel samples were analyzed by Raman spectroscopy. The typical rGO spectrum is featured by the presence of four main vibrational modes, namely: D, G, 2D and D+G (D+D’) [43]. In graphite-like materials, the G mode is related to the organized carbon hexagonal structure, while the D mode is related both to the amount of defects in hexagonal graphene sheets and the number of functional groups (or doping). The overall quality of GO samples can be estimated from the D to G mode intensity ratio (*I*_D_/*I*_G_). The *I*_D_/*I*_G_ ratios of the synthesized GO powder and the rGO aerogel, estimated from Raman spectra (data not shown) were 0.9 and 1.05, respectively. The *I*_D_/*I*_G_ ratio increase is related to removal of oxygen functional groups and the decrease of the average size of the sp^2^ domains upon hydrothermal reduction [44–47]. Figure 3 presents spectra of rGO, rGO-Fe_3_O_4_ and rGO-PDA@Fe_3_O_4_ aerogel samples obtained at different wavelengths: λ = 488 nm (*E*_L_ = 2.54 eV), λ = 514 nm (*E*_L_ = 2.41 eV), λ = 633 nm (*E*_L_ = 1.96 eV) and λ = 785 nm (*E*_L_ = 1.58 eV). In this figure one can notice typical rGO Raman vibrational response with different intensities together with modes related to Fe_3_O_4_. The polydopamine and PDA@Fe_3_O_4_ spectra are shown in Figure S2 of Supporting Information File 1. Polydopamine exhibits Raman response in similar region to rGO. However, due to the relative low concentration of PDA in the samples, it do not interfere with recorded spectra. The analysis of Raman spectra of rGO-based structures using single wavelength can lead to false conclusions. The spectra obtained using laser with excitation energy *E*_L_ = 1.58 eV present high *I*_D_/*I*_G_ ratio with low intensity of 2D and D+G modes, which could suggest that the observed material is an amorphous carbon. However, the use of other excitation energies leads to completely different conclusions. The calculated *I*_D_/*I*_G_ ratios and mean defect distance (*L*_D_) together with full-widths-at-half-maximum (FWHM) of D and G peaks of rGO-based aerogels probed with different laser sources are presented in Figure 4.

The analysis of changes in *I*_D_/*I*_G_ ratio (Figure 4a) shows two effects: (i) regardless of the sample, the *I*_D_/*I*_G_ ratio decreases with the increase of applied excitation energy and (ii) the *I*_D_/*I*_G_ ratio decreases after the addition of magnetite nanoparticles. Therefore, in comparison to reference rGO aerogel, the rGO-Fe_3_O_4_ and rGO-PDA@Fe_3_O_4_ aerogel structures have less defects and higher structural order. The first effect is related to a k-selective resonant Raman scattering process observed in graphite-based materials, where the defect-activated D mode is excitation laser-dependent due to a double resonance process [48]. This effect is also confirmed by the 33 cm^−1^ D mode blue-shift in the rGO-Fe_3_O_4_ spectrum obtained with highest excitation energy *E*_L_ = 2.54 eV. The second effect, i.e., the decreased *I*_D_/*I*_G_ ratio of modified aerogel samples as compared to the ratio obtained for the reference rGO aerogel, is related to the stabilization of rGO aerogel structure by Fe_3_O_4_ nanoparticles that anchor at the GO defect sites via monodente or bidentate coordination of iron with carboxylic groups during hydrogel formation [49]. In the case of the rGO-PDA@Fe_3_O_4_ sample, this effect is even more pronounced. The 6 cm^−1^ red-shift of the G mode observed in rGO-PDA@Fe_3_O_4_ spectra obtained at λ = 514 nm and λ = 785 nm also confirm this assumption, as it is known that G mode shifts towards lower frequencies along with the decrease of the number of defects and subsequent formation of sp^2^ clusters in defective carbon lattices [50]. The *L*_D_ parameter is dependent on *E*_L_ and *I*_D_/*I*_G_ ratio and changes along with defects concentration. The mean defect distance can be estimated from the following equation [51]:

[1]
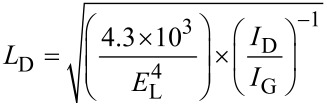


From the analysis of the *L*_D_ one can obtain information related to the degree of amorphization of graphene. This process can be divided into two stages, as described in ref. [51]. It can be seen from Figure 4b, that the *L*_D_ in both samples is higher than 3 nm regardless of excitation energy. Thus, it can be deduced that the investigated rGO, rGO-Fe_3_O_4_ and rGO-PDA@Fe_3_O_4_ aerogel samples are “stage 1” defected graphene with largely intact honeycomb lattice and carbon domains that contain at least 300 atoms [51–53]. Interestingly, the mean defect distance increases along with the decrease of the *I*_D_/*I*_G_ ratio and increase of the structural order – which is particularly pronounced in the case of the rGO-PDA@Fe_3_O_4_ sample. This effect confirms that the polydopamine-related carbon atoms are used for structural reorganization of the rGO hexagonal lattice. It is assumed, that this effect could be improved by increasing the polydopamine content. In Figure 4c one can observe changes of FWHM_D_ and FWHM_G_ in the Raman spectra of MNPs-modified aerogel samples compared to the rGO reference. The measurements of the rGO-Fe_3_O_4_ sample performed with *E*_L_ = 2.54 eV revealed the decrease of FWHM_D_ and FWHM_G_ by 22.05 cm^−1^ and 10.03 cm^−1^, respectively. It is believed that this effect is caused by the increase of the lattice parameter and decrease of the C–C bond strength due to anchored Fe_3_O_4_ nanoparticles or Fe doping at the defect sites. At lower excitation energies, the FWHM_G_ values were comparable to those of rGO reference sample (within the range of the measurement error). The FWHM_D_ values differed along with the changing energies of laser excitation which confirmed that the D mode is excitation laser-dependent. An exception were measurements performed with *E*_L_ = 1.96 eV, whereas FWHM of both D and G modes of MNPs-modified aerogel samples did not increase more than 2.5 cm^−1^ with respect to the rGO reference value.

**Figure 5:**
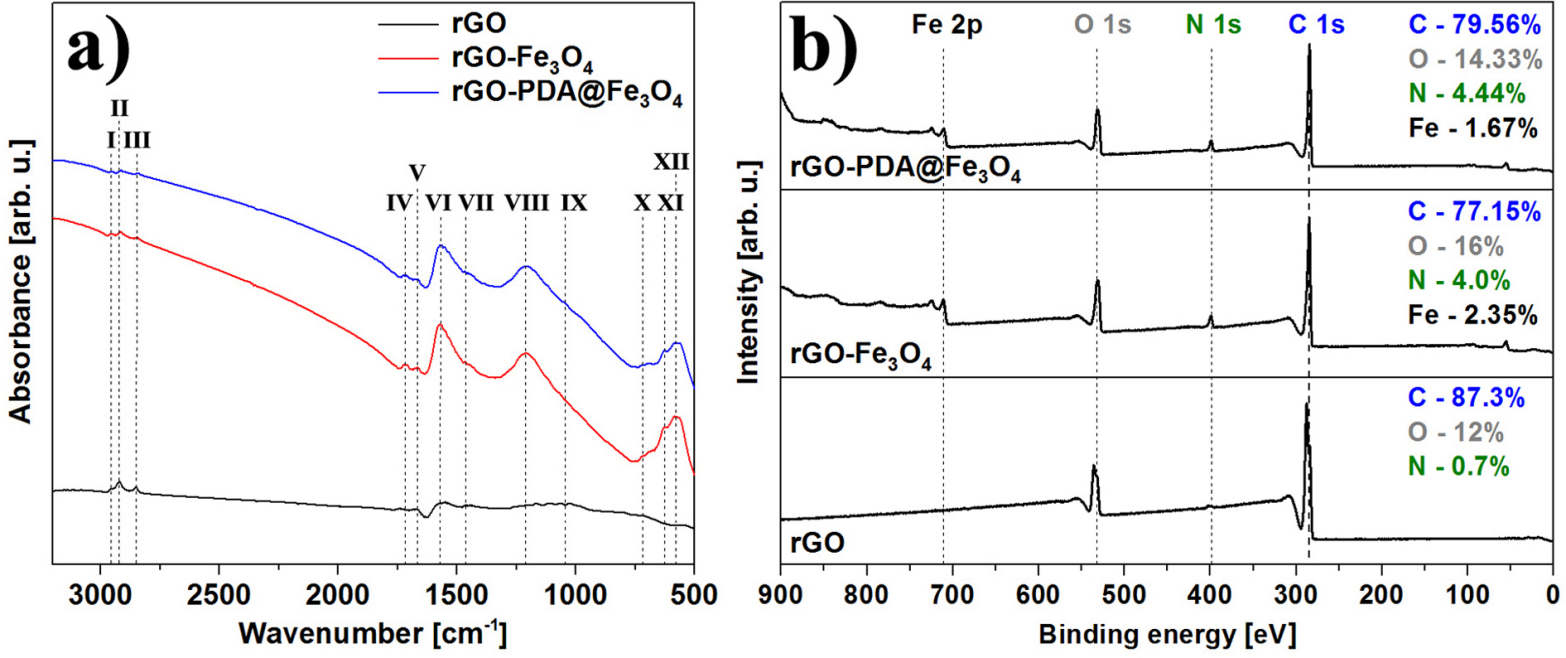
FTIR absorption (a) and XPS survey (b) spectra of rGO, rGO-Fe_3_O_4_ and rGO-PDA@Fe_3_O_4_ aerogel samples.

In order to investigate the nature of possible functional groups at the aerogels surface, the samples were analyzed using FTIR spectroscopy. Figure 5a presents FTIR spectra obtained for rGO, rGO-Fe_3_O_4_ and rGO-PDA@Fe_3_O_4_ aerogels. From this figure one can notice the difference in intensity of ≈2959 cm^−1^ (I), 2921 cm^−1^ (II) and 2849 cm^−1^ (III) bands, corresponding to rGO and rGO-MNPs ν_as_ CH_3_ and ν_as_/ν_s_ CH_2_ stretching, respectively. This effect could be related to the defect sites termination modified during the hydrothermal synthesis process. In the case of MNP hybrid structures, the intensity decreased due to anchoring of Fe_3_O_4_ nanoparticles at the defect sites. All the spectra are featured by the C=C aromatic double bond at 1667 cm^−1^ (V) and the C–C bond at 1447 cm^−1^ (VII). However, only hybrid aerogels exhibit C=O and C–O vibrations at 1723 cm^−1^ (IV) and 1205 cm^−1^ (VIII), respectively, which may originate from the presence of citric acid or polydopamine. The residual ether-type functional groups can be observed via C–O vibrational band at 1115 cm^−1^ (IX). The strong band at 1572 cm^−1^ (VI) could be attributed to a carboxylate C–O–Fe bond, which confirms covalent bonding between magnetite surface and CA/PDA coating, as well as lack of free -OH groups. Low frequency bands at 691 cm^−1^ (X), 626 cm^−1^ (XI) and 570 cm^−1^ (XII) are related to Fe–O vibrations in Fe_3_O_4_ nanoparticles. Interestingly, the C–N and N–H vibrations related to the presence of PDA, previously spotted on the spectrum of PDA@Fe_3_O_4_ nanoparticles (Figure S3 in Supporting Information File 1), were not observed for the aerogel sample [38].

**Figure 6:**
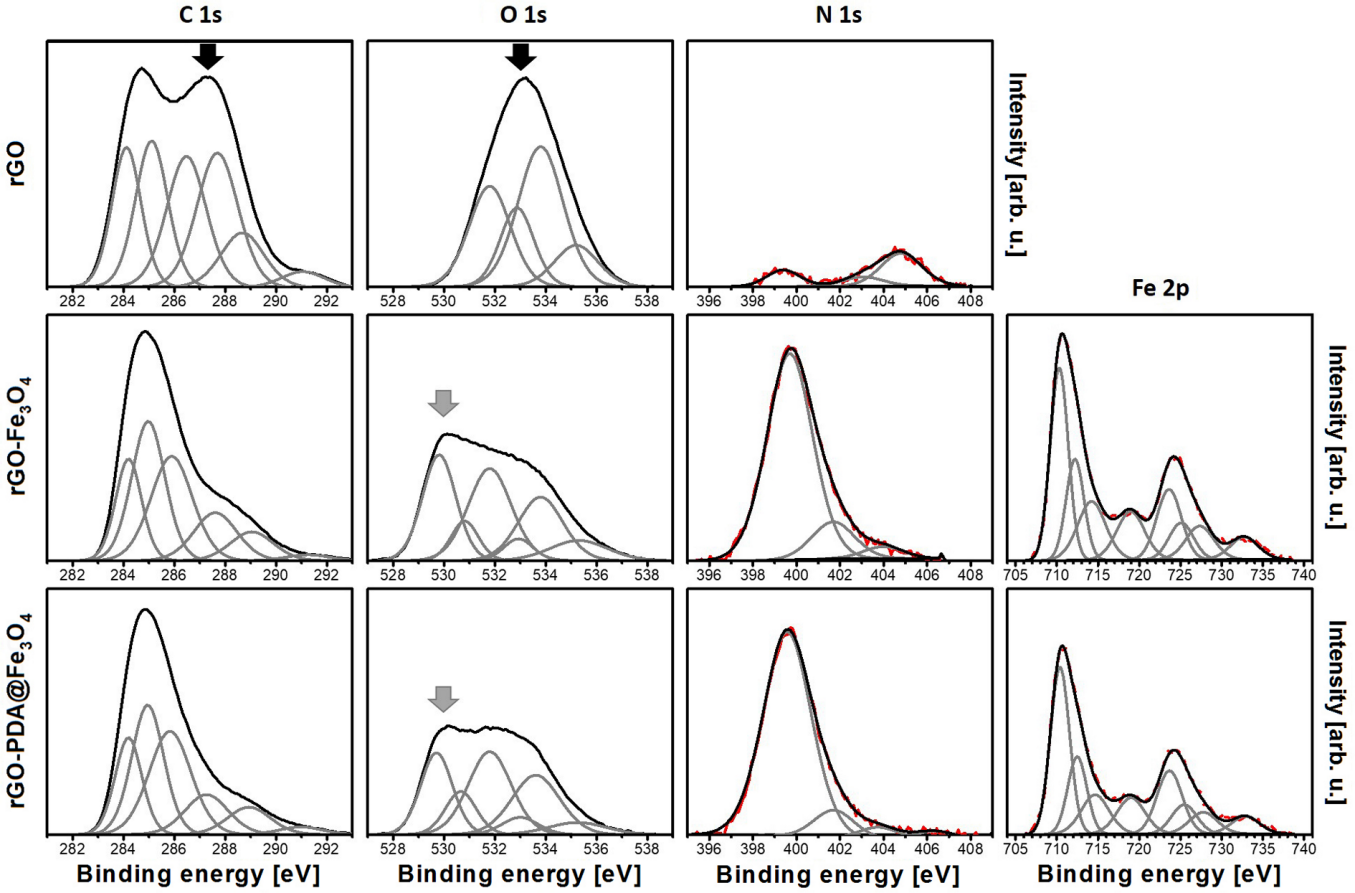
XPS C 1s, O 1s, N 1s and Fe 2p spectra of rGO, rGO-Fe_3_O_4_ and rGO-PDA@Fe_3_O_4_ aerogel samples. The intensity scales are the same for respective elements (in order to allow for direct comparison).

The chemical composition of the samples was studied using XPS. The survey spectra, shown in Figure 5b, revealed the presence of C, O and N (in all the samples), as well as Fe (in MNPs containing samples). Other elements, if present, were beyond the detection limit of the instrument. The low amount of oxygen in the reference sample confirmed efficient GO reduction during hydrogel formation. Differences in C concentration in rGO@Fe_3_O_4_ and rGO-PDA@Fe_3_O_4_ samples were assigned to the addition of carbon-containing polydopamine coating, while differences in the observed amount of Fe were rationalized in terms of surface sensitivity of XPS, resulting in a higher Fe signal when probing Fe_3_O_4_ directly and a lower signal when probing the oxide through a PDA coating. The presence of nitrogen in the rGO-PDA@Fe_3_O_4_ sample was expected, as PDA contains amine groups. The nitrogen signal in the rGO sample was similar to that of the reference GO (Figure S4 in Supporting Information File 1) and assigned to originate from the GO synthesis process. The increase in nitrogen in the rGO-Fe_3_O_4_ sample was in turn related to ammonia used for magnetite nanoparticle synthesis. The deconvoluted detailed C 1s, O 1s, N 1s and Fe 2p spectra are presented in Figure 6. The positions and concentrations of the fitted peaks are listed in Table S1 in Supporting Information File 1. The C 1s lines were fitted with six components. The two peaks positioned at lowest binding energies were assigned to C–C and C=C bonded carbon [54]. The next three are believed to originate from carbon atoms in various surface functional groups, such as C–O, C–N, C=O and O=C–OH [55]. The highest binding energy component was assigned to originate from adsorbed carbon-containing molecules, such as CO or CO_2_. The O 1s spectra were also fitted with several components originating from oxygen in the above-mentioned functional groups and adsorbed oxygen-containing molecules (in the case of all samples) [56–57], as well as from iron oxide (in the case of MNPs containing samples) [58]. Interestingly, the presence of quinone groups was observed in rGO-Fe_3_O_4_ and rGO-PDA@Fe_3_O_4_ samples. These groups do not exhibit peaks in the C 1s region which could allow distinguishing them from other carbon-containing groups [57], therefore, they could be only identified from the analysis of the oxygen peak. The N 1s regions were fitted with several components that are believed to correspond to pyridinic (N–C), pyrrolic (N–H), graphitic, oxidized and chemisorbed nitrogen [41,59]. Unfortunately, the high amount of nitrogen originating from ammonia in Fe_3_O_4_-containing samples made the identification of the PDA-related nitrogen peaks problematic.

The analysis of the iron Fe 2p region indicated the presence of Fe_3_O_4_, as the fitted components – corresponding to Fe^2+^, Fe^3+^ and satellites – were positioned at the characteristic binding energy values [60]. The most important observation was that addition of Fe_3_O_4_ and PDA@Fe_3_O_4_ nanoparticles results in the decrease of the C 1s and O 1s signals corresponding to C=O and O=C–OH functional groups (marked with black arrows in Figure 6), which is accompanied by an increase of the O 1s component corresponding to metal oxide (grey arrows). This indicate that Fe_3_O_4_ and PDA@Fe_3_O_4_ nanoparticles are replacing the above mentioned functional groups and attach at the defects sites at the graphene lattice. In that way, the defects act as anchoring centers for the particles. This assignment was further confirmed by the decrease of the C–O component in the O 1s spectra of the rGO-Fe_3_O_4_ and rGO-PDA@Fe_3_O_4_ samples. Therefore, it was concluded that the nanoparticles attach to the aerogel replacing C=O, O=C–OH and C–O groups.

**Figure 7:**
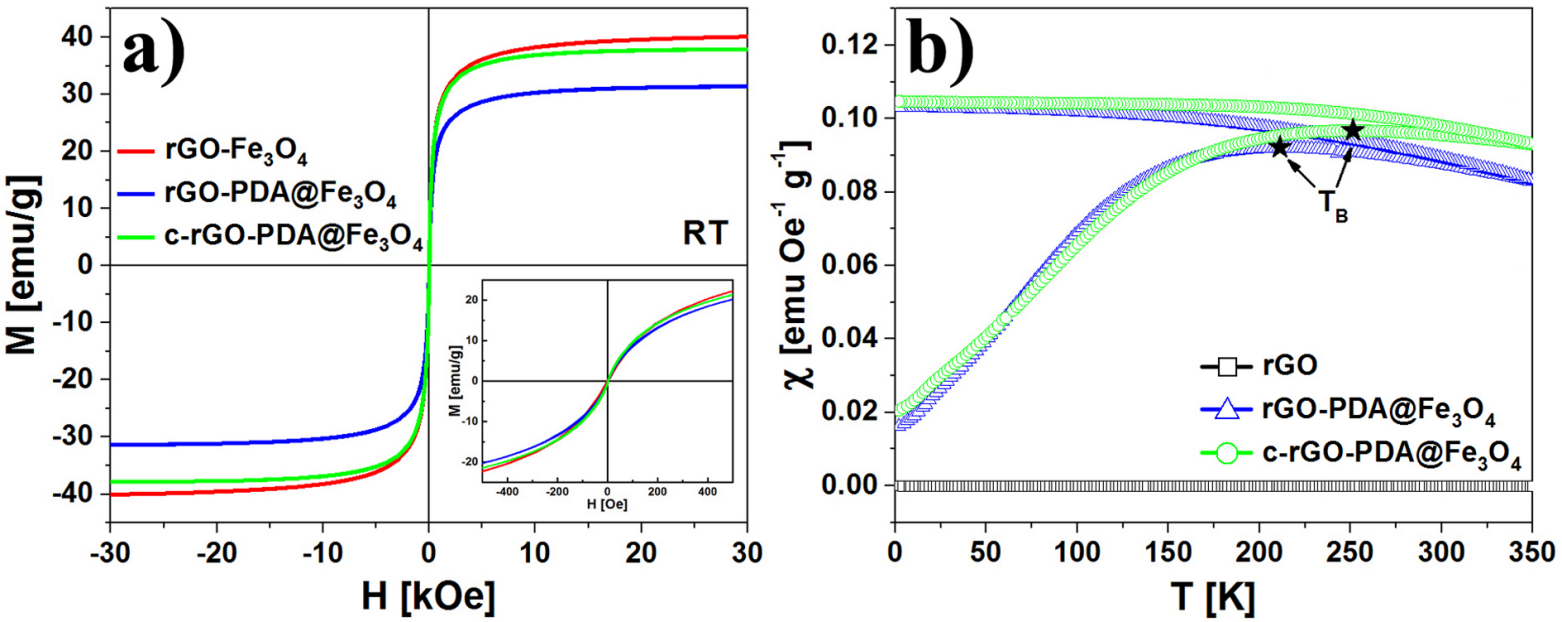
Room temperature M–H curves of rGO-Fe_3_O_4_, rGO-PDA@Fe_3_O_4_ and c-rGO-PDA@Fe_3_O_4_ aerogels (a). The inset in (a) presents M–H curves at low magnetic field. ZFC and FC temperature dependences of susceptibility for rGO, rGO-PDA@Fe_3_O_4_ and c-rGO-PDA@Fe_3_O_4_ aerogels under the applied field of 100 Oe are shown in (b).

The magnetic properties of the reference and Fe_3_O_4_-modified rGO aerogels were investigated by performing magnetic susceptibility measurements at various temperatures. Part of the rGO-PDA@Fe_3_O_4_ sample was additionally compressed in casting die (10 tons) in order to determine the influence of nanoparticles distribution (distances between agglomerates) on the magnetic response in porous and compressed aerogel structures (this sample is further referred as c-rGO-PDA@Fe_3_O_4_). The magnetization curves are presented in Figure 7.

The room temperature magnetization dependences on the magnetic field (M–H curves) for rGO-Fe_3_O_4_, rGO-PDA@Fe_3_O_4_ and c-rGO-PDA@Fe_3_O_4_ aerogels are shown in Figure 7a. It can be noticed that all the M–H curves have non-hysteretic, superparamagnetic-like character. The saturation magnetization was found to be 38.2 emu/g, 30.5 emu/g and 36.9 emu/g for rGO-Fe_3_O_4_, rGO-PDA@Fe_3_O_4_ and c-rGO-PDA@Fe_3_O_4_ aerogel samples, respectively. It was generally expected that covering the Fe_3_O_4_ nanoparticles with an organic (PDA) shell will increase the magnetization of the samples [61], thanks to the protection of the magnetite surface from an environmental oxidation [62–64]. However, the saturation magnetization of rGO-PDA@Fe_3_O_4_ with respect to the rGO-Fe_3_O_4_ was decreased, which is due to additional contribution of PDA to the sample volume [65]. The agglomeration of magnetic nanoparticles has a direct effect on any measurement performed on NPs when extracting quantitative parameters, such as, e.g., the magnetic moment value [66]. The interparticle distance affects the saturation magnetization of magnetic nanoparticles, as the strength of the magnetic moment interaction depends on the interparticle distance [67]. Therefore, the compression of nanoparticles in the c-rGO-PDA@Fe_3_O_4_ aerogel sample led to the increase of the saturation magnetization. Figure 7b presents ZFC and FC susceptibility curves obtained for rGO, rGO-PDA@Fe_3_O_4_ and c-rGO-PDA@Fe_3_O_4_ aerogel samples. The susceptibility of the rGO aerogel has an insignificant contribution (10^−6^ emu Oe^−1^ g^−1^) to the susceptibility of Fe_3_O_4_-modified GO aerogels. In the case of magnetite containing aerogels, both ZFC and FC susceptibility curves coincide at high temperatures and separate as the temperature is decreasing. The maximum of the ZFC curve is defined as the blocking temperature (TB) of the superparamagnetic nanoparticles and the width of the ZFC curve maximum is related to the size distribution or agglomeration process of superparamagnetic nanoparticles. The TB determined for the rGO-Fe_3_O_4_ (Figure S5 in Supporting Information File 1) and rGO-PDA@Fe_3_O_4_ aerogels is very similar – in the range of 215 K < TB < 220 K. This indicates negligible influence of PDA on the magnetic properties of Fe_3_O_4_ nanoparticles. The compression of rGO-PDA@Fe_3_O_4_ aerogel led to the increase of TB to 252 K. This could be caused by several biased effects: (i) an increase of the sample density, (ii) a decrease of the distance between magnetite nanoparticles and (iii) an increase of dipole–dipole interactions between them [68].

## Conclusion

The influence of polydopamine coating of magnetite nanoparticles on the structure and properties of rGO-Fe_3_O_4_ aerogels was studied. It was found that the polydopamine coating has a positive effect on the aerogel structure by supplying carbon atoms to the defected hexagonal structure and anchoring of PDA-coated Fe_3_O_4_ nanoparticles at the GO defect sites. In addition, PDA coating does not affect the magnetic properties of the iron oxide-modified rGO aerogel. It is believed that introduction of amorphous carbon-coated functional additives (core-shell structures) improves a reduced graphene oxide aerogel lattice, anchor functional additives at the rGO defect sites, prevent unintended additives migration outside the aerogel and provide better structural stabilization of the whole aerogel structure.

## Experimental

Graphite powder, iron(III) chloride hexahydrate, iron(II) chloride tetrahydrate, sodium nitride 99%, citric acid ≥99.5%, dopamine hydrochloride and ethylenediamine were obtained from Sigma-Aldrich. Hydrochloric acid 35–38%, sulfuric acid 95%, ethanol 99.8%, ethanol 96% and hydrogen peroxide solution 30% were purchased from POCH. Ammonia solution 25% and potassium permanganate were obtained from Chempur and J.T. Baker, respectively. All chemical reagents were of analytical grade and used as received. All water solutions were based on deionized water (DI-H_2_O).

Graphene oxide was prepared using the modified Hummer’s method from commercial graphite powder [69]. Briefly, 1 g of graphite and 0.5 g of NaNO_3_ were mixed together followed by the addition of 23 mL of 95% H_2_SO_4_ under constant stirring for 1 hour. Then, 3 g of KMnO_4_ were added gradually to the solution at a temperature below 20 °C to prevent the risk of overheat and explosion. Following this, the mixture was stirred at 35 °C for 12 hours. Next, it was diluted with 500 mL of DI-H_2_O under stirring and 4.6 mL of 30% H_2_O_2_ was added to complete the reaction. The as prepared mixture was washed with HCl and DI-H_2_O and purified via multiple ultrasonication/ultracentrifugation cycles in DI-H_2_O. Finally, samples were centrifuged in 96% C_2_H_5_OH, decanted, collected and dried overnight at 80 °C.

Magnetite nanoparticles were synthesized via a co-precipitation process, as follows: FeCl_2_·4H_2_O (1.72 g, 8.65 mmol) and FeCl_3_·6H_2_O (4.7 g, 17.38 mmol) were dissolved in water and degassed. Next, the temperature was elevated to 85 °C and 25% ammonia solution (20 mL) was added under vigorous stirring. After 30 minutes, 8 mL of citric acid were added. The process was continued at 95 °C for 90 minutes. Subsequently, the mixture was cooled down to room temperature (RT) and the obtained nanoparticles were washed with DI-H_2_O (3 × 200 mL) and finally dispersed in 100 mL of DI-H_2_O. Modification of Fe_3_O_4_ nanoparticles with polydopamine from dopamine hydrochloride was performed in the same manner as described in ref. [38]. Citric acid and citric acid-polydopamine-coated Fe_3_O_4_ nanoparticles are referred to as “Fe_3_O_4_” and “PDA@Fe_3_O_4_”, respectively.

Three DI-H_2_O solutions of ultrasound dispersed GO (2 mg/mL), GO (2 mg/mL) with Fe_3_O_4_ (1 mg/mL) and GO (2 mg/mL) with PDA@Fe_3_O_4_ (1 mg/mL) were prepared in 10 mL glass beakers. Then 60 μL of ethylenediamine were added as a reduction agent to each beaker. Next, the beakers were placed in a Teflon-metal covered autoclave and held in furnace at 180 °C (ramp 10 °C/min) for 2 hours. During hydrogel formation, the GO undergoes reduction. Therefore, further GO hydro-, alco- and aerogels are called reduced GO (rGO). As obtained rGO hydrogels underwent solvent exchange with subsequent 25%, 50%, 75%, 96%, 99.8% ethanol solutions for 4, 4, 4, 8 and 12 hours, respectively. Each of the prepared rGO alcogels was cut and placed in a critical point dryer (CPD). The liquid CO_2_ was exchanged every 2 hours during the 8 hour process. Subsequently, the temperature was increased to 35 °C and the pressure to 1200 psi. Finally, the CPD chamber was left overnight with slightly open valve for gentle depressurization. As obtained rGO aerogel samples, signed as “rGO” (reference), “rGO-Fe_3_O_4_” and “rGO-PDA@Fe_3_O_4_”, were stored for further analysis.

The morphology of the aerogels was analyzed by scanning electron microscopy (SEM) (FEI, NovaNanoSEM 650). The diameter distribution of magnetite nanoparticles was determined using transmission electron microscopy (TEM) (Jeol, 1400, 120 kV). The vibrational properties of the investigated samples were examined by micro-Raman spectroscopy (Renishaw, inVia Raman microscope) operating at λ = 785 nm, 633 nm, 514 nm and 488 nm wavelengths. For each sample, the measurements were performed at three different spots. All the spectra were subtracted to straight-line from 500 cm^−1^ to 3100 cm^−1^ and normalized to the graphene G mode. The functional groups in the prepared aerogels were investigated using Fourier Transform Infrared (FTIR) spectroscopy (Bruker Optics, TENSOR 27) equipped with a MCT detector and globar source. The chemical composition of the samples was studied using X-ray photoelectron spectroscopy (XPS). The measurements were performed in an ultra-high vacuum (UHV) chamber using a monochromatic Al Kα X-ray source (Omicron, XM1000) and a semispherical electron energy analyzer (Omicron, SPHERA II) operating at pass energies of 50 (survey spectra) and 20 eV (regions). The data were calibrated with respect to the Fermi level of the sample and fitted using the CasaXPS software (Casa Software Limited). The magnetic properties of Fe_3_O_4_-containing samples were studies using MPMS-XL SQUID magnetometer (Quantum Design) by performing susceptibility and magnetization reversal measurements. Zero- (ZFC) and field-cooled (FC) susceptibility curves were obtained at 100 Oe with temperature varying from 2 to 350 K. The magnetic hysteresis loops (M–H dependences) were measured at RT at a magnetic field varying between ±30 kOe. Powder X-ray diffraction (XRD) studies of source materials were carried out to determine the crystallographic structure of the studies compounds. The diffractometer (PANalytical, Empyrean) was equipped with a Cu Kα (1.54 Å) radiation source, reflection-transmission spinner (sample stage) and a PIXcel 3D detector (operating in the Bragg-Brentano geometry).

## Supporting Information

Supporting Information contains XRD patterns of graphite, graphene oxide and Fe_3_O_4_ nanoparticles, Raman spectra of PDA and PDA@Fe_3_O_4_ nanoparticles, FTIR spectrum of PDA@Fe_3_O_4_ nanoparticles, XPS survey spectrum and deconvoluted C 1s, O 1s and N 1s spectra of graphene oxide, ZFC and FC temperature dependences of magnetic susceptibility for rGO-Fe_3_O_4_ aerogel under the applied field of 100 Oe, Table with XPS peak assignments, positions (eV) and percentage contributions (%).

File 1 Additional information.
