# Supplementary material for: Anchoring Fe3O4 nanoparticles in a reduced graphene oxide aerogel matrix via polydopamine coating
Source: Beilstein J Nanotechnol. 2018 Feb 15;9:591–601. doi: 10.3762/bjnano.9.55 (PMC5827803; doi:10.3762/bjnano.9.55)
Supplement: File 1 — Additional information. [file Beilstein_J_Nanotechnol-09-591-s001.pdf]

Supporting Information

for

Anchoring Fe_3O_4 nanoparticles in a reduced graphene oxide aerogel matrix via polydopamine coating

Błażej Scheibe[*1], Radosław Mrówczyński[1], Natalia Michalak[2], Karol Załęski[1], Michał Matczak[2], Mateusz Kempiński[1,3], Zuzanna Pietralik[3], Mikołaj Lewandowski[1], Stefan Jurga[1] and Feliks Stobiecki[1,2]

Address: [1]NanoBioMedical Centre, Adam Mickiewicz University, Umultowska 85, 61-614 Poznań, Poland, [2]Institute of Molecular Physics, Polish Academy of Sciences, M. Smoluchowskiego 17, 60-179 Poznań, Poland and [3]Faculty of Physics, Adam Mickiewicz University, Umultowska 85, 61-614 Poznań, Poland

Email: Błażej Scheibe - bscheibe@amu.edu.pl

* Corresponding author

Additional information

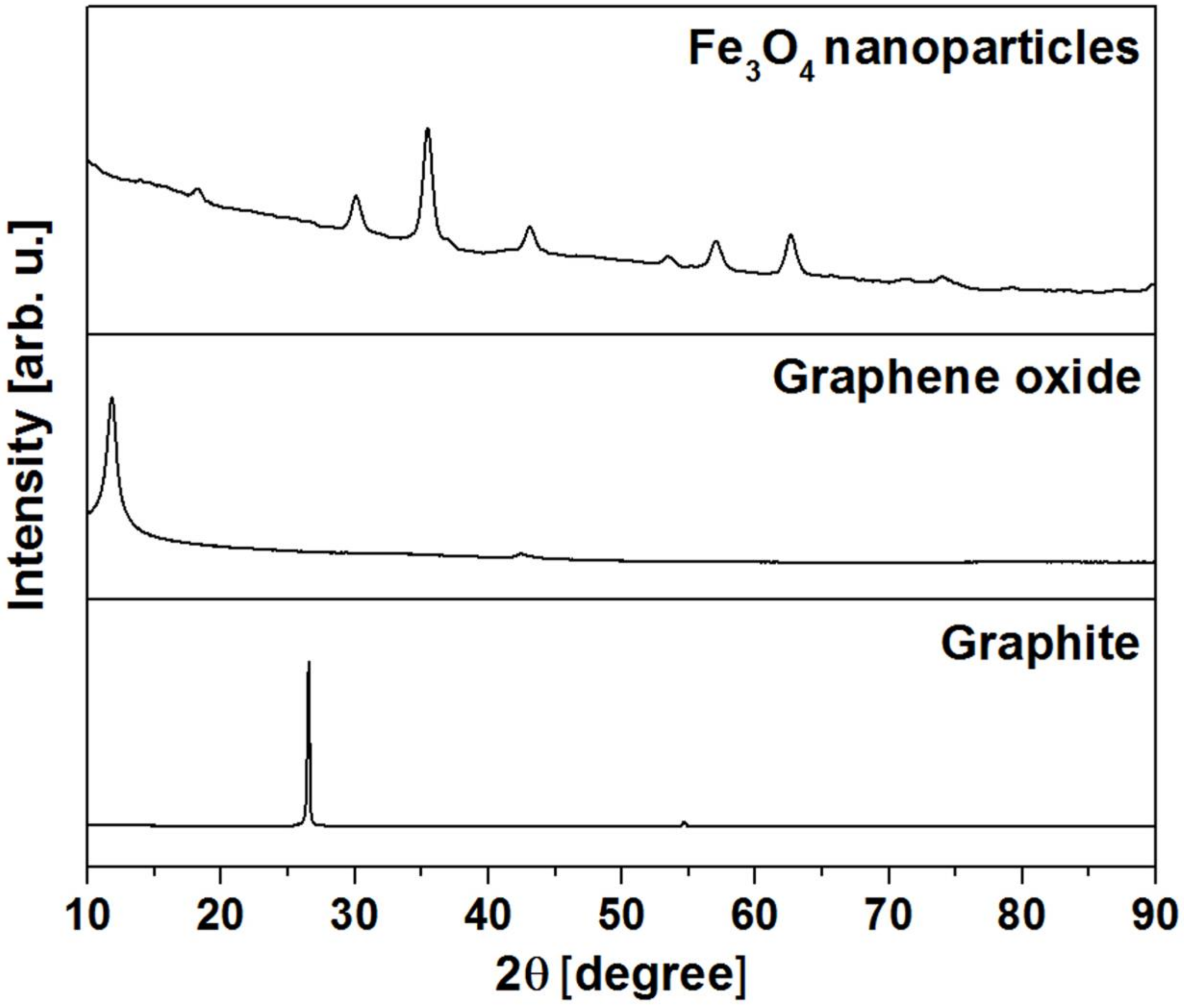

Figure S1: XRD patterns of graphite, graphene oxide and Fe_3O_4 nanoparticles.

The XRD patterns of composite aerogels were dominated by Fe_3O_4 peaks (data not shown).

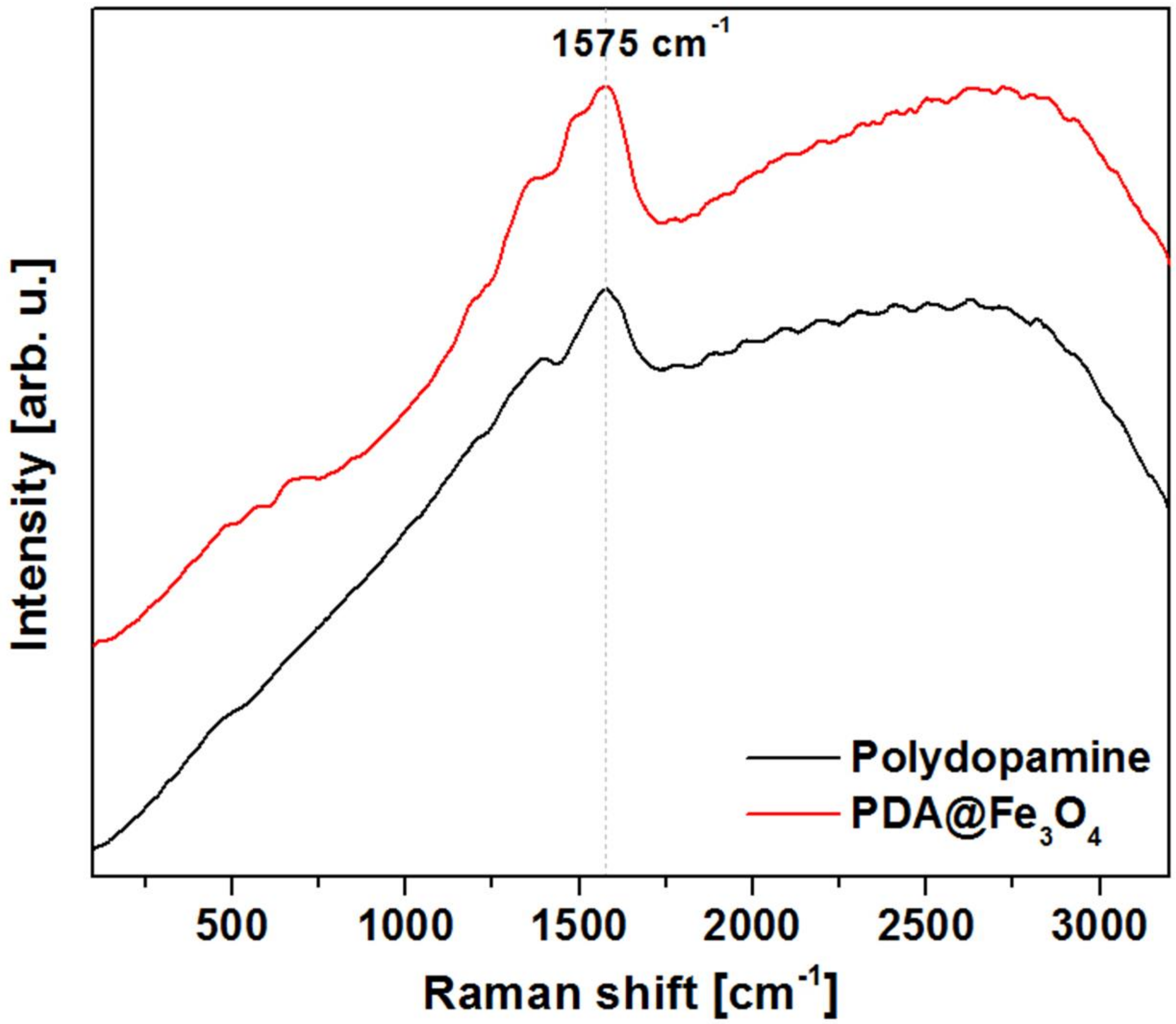

Figure S2: Raman spectra of PDA and PDA@Fe_3O_4 nanoparticles.

The highest peak at 1575 cm^{-1} does not overlap with the G-mode of RGO at 1591 cm^{-1}.

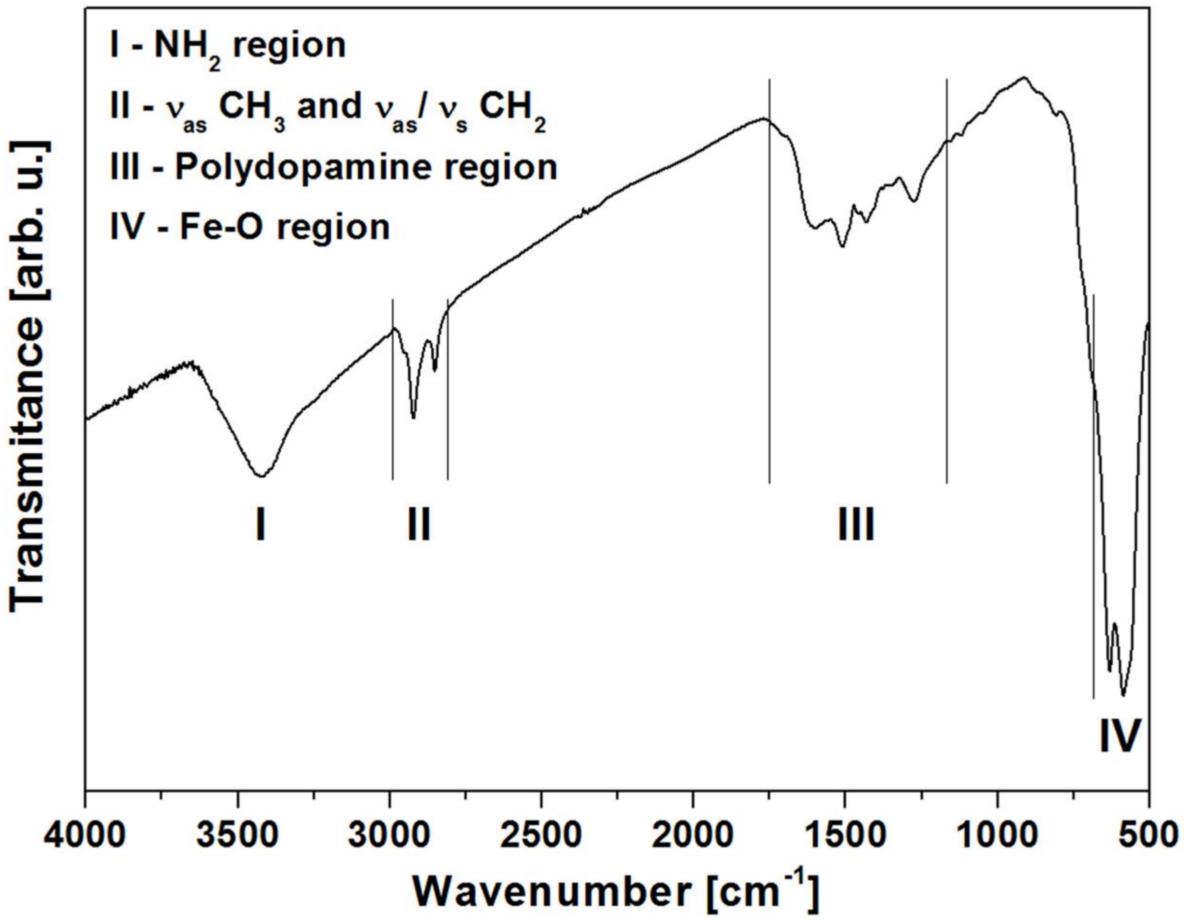

Figure S3: FTIR spectrum of PDA@Fe_3O_4 nanoparticles.

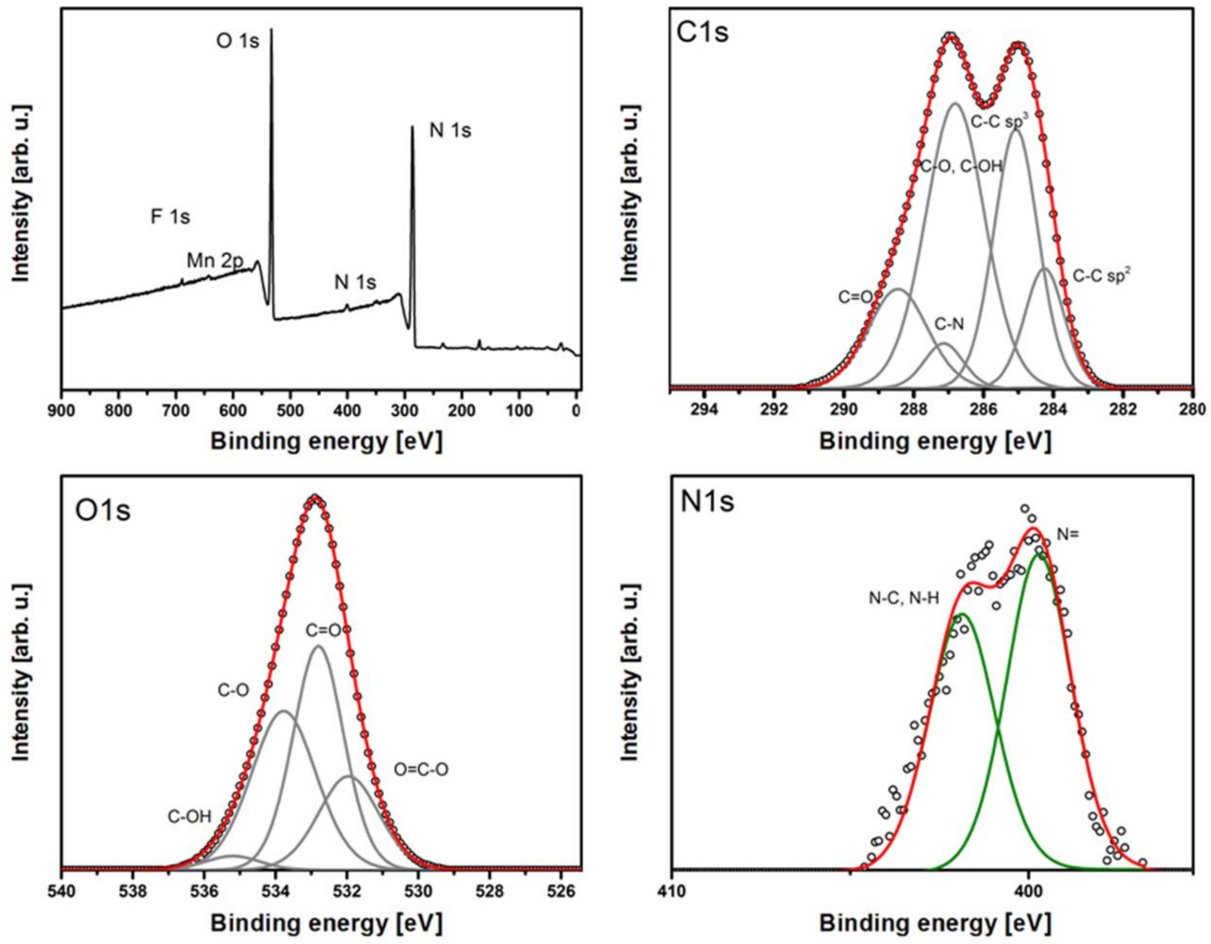

Figure S4: XPS survey spectrum and deconvoluted C 1s, O 1s and N 1s spectra of graphene oxide.

C-74.6%, O-24%, N-1%, F-0.3%, Mn-0.1%

C-C sp^2 : 284.3 eV   C-C sp^3 : 285.1 eV   C-N : 287.1 eV   C-O, C-OH : 286.8 eV

C=O : 288.5 eV

O=C-OH : 531.9 eV   C=O : 532.8 eV   C-O : 533.7 eV   C-OH : 535.2 eV

N= : 399.7 eV   N-C, N-H : 401.8 eV

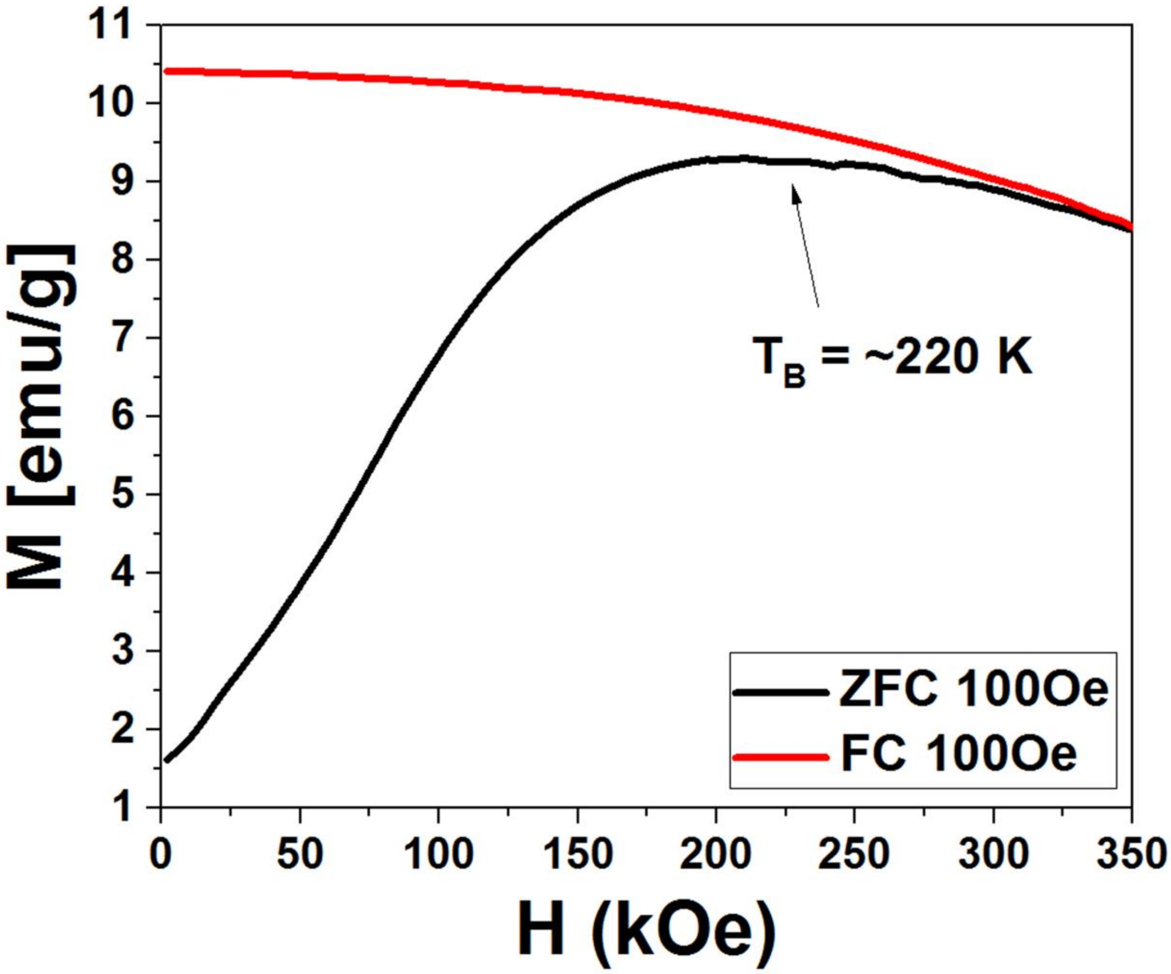

Figure S5: ZFC and FC temperature dependences of magnetic susceptibility for rGO-Fe_3O_4 aerogel under the applied field of 100 Oe.

Table S1: XPS peak assignments, positions (eV) and percentage contribution (%).

**C 1s spectrum**	Adsorbed molecules (CO, CO_2)		Functional groups						C-C *sp*^{3}		C-C *sp*^{2}	
	eV	%	eV	%	eV	%	eV	%	eV	%	eV	%
rGO	291.0	3	288.7 (O=C-O)	10	287.7 (C=O)	25	286.5 (C-O)	23	285.1	21	284.12	18
rGO-Fe_3O_4	291.2	2	289 (O=C-O)	8	287.6 (C=O)	14	286.0 (C-N)	29	285.0	30	284.2	17
rGO-PDA@Fe_3O_4	291.0	2	289 (O=C-O)	8	287.3 (C=O)	12	285.9 (C-N)	30	284.9	29	284.2	18

**O 1s spectrum**	Adsorbed molecules (O_2, H_2O, CO, CO_2)		Functional groups						Metal oxide	
	eV	%	eV	%	eV	%	eV	%	eV	%
rGO	-	-	534.7	27	533.4 (O=C-O)	34	532.0 (C-O)	38	-	-
rGO-Fe_3O_4	535.8	5	534.1 O=C-O	18	532.7 (C-O)	23	531.1 (C=O)	31	529.7	22
rGO-PDA@Fe_3O_4	535.4	6	533.6 (O=C-O)	24	532.3 (C-O)	22	531.0 (C=O)	26	529.7	22

**N 1s spectrum**	Adsorbed molecules		Graphitic and oxidized nitrogen				Pyridinic and pyrrolic nitrogen	
	eV	%	eV	%	eV	%	eV	%
rGO	-	-	404.7	46	402.7	18	399.3	36
rGO-Fe_3O_4	-	-	404.0	5	401.7	13	399.7	82
rGO-PDA@Fe_3O_4	406.3	3	403.7	4	401.7	10	399.6	83

**Fe 2p spectrum**	2p $_{1/2}$ Fe^{3+}		2p $_{1/2}$ Fe^{2+}		2p $_{3/2}$ Fe^{3+}		2p $_{3/2}$ Fe^{2+}	
	eV	%	eV	%	eV	%	eV	%
rGO-Fe_3O_4	725.4	17	723.6	32	712.2	17	710.3	33
rGO-PDA@Fe_3O_4	725.5	16	723.6	34	712.5	16	710.4	35
